# Decentralization of the health system – experiences from Pakistan, Portugal and Brazil

**DOI:** 10.1186/s12961-024-01145-3

**Published:** 2024-05-27

**Authors:** Shafaq Mahmood, Rita Sequeira, Muhammad Muneeb Ullah Siddiqui, Marcos Batista Araujo Herkenhoff, Patrícia Pita Ferreira, Adalberto Campos Fernandes, Paulo Sousa

**Affiliations:** 1 Community Health Sciences, The Aga Khan University, Karachi, Pakistan; 2 NOVA National School of Public Health, Public Health Research Centre, Comprehensive Health Research Center (CHRC), NOVA University Lisbon, Lisbon, Portugal; 3 Federal University of Espirito Santo, Vitoria, ES Brazil; 4 Unidade de Saúde Pública Zé Povinho, ACES Oeste Norte, Caldas da Rainha, Portugal

**Keywords:** Decentralization, Health sector reform, Performance of health systems, LMICs, Developed country

## Abstract

**Background:**

Decentralization of a health system is a complex and multidimensional phenomenon that demands thorough investigation of its process logistics, predisposing factors and implementation mechanisms, within the broader socio-political environment of each nation. Despite its wide adoption across both high-income countries (HICs) and low-and-middle-income countries (LMICs), empirical evidence of whether decentralization actually translates into improved health system performance remains inconclusive and controversial. This paper aims to provide a comprehensive description of the decentralization processes in three countries at different stages of their decentralization strategies – Pakistan, Brazil and Portugal.

**Main body:**

This study employed a systematic analysis of peer-reviewed academic journals, official government reports, policy documents and publications from international organizations related to health system decentralization. A comprehensive search was conducted using reputable databases such as PubMed, Google Scholar, the WHO repository and other relevant databases, covering the period up to the knowledge cutoff date in June 2023. Information was systematically extracted and organized into the determinants, process mechanics and challenges encountered during the planning, implementation and post-decentralization phases. Although decentralization reforms have achieved some success, challenges persist in their implementation. Comparing all three countries, it was evident that all three have prioritized health in their decentralization reforms and aimed to enhance local decision-making power. Brazil has made significant progress in implementing decentralization reforms, while Portugal and Pakistan are still in the process. Pakistan has faced significant implementation challenges, including capacity-building, resource allocation, resistance to change and inequity in access to care. Brazil and Portugal have also faced challenges, but to a lesser extent. The extent, progress and challenges in the decentralization processes vary among the three countries, each requiring ongoing evaluation and improvement to achieve the desired outcomes.

**Conclusion:**

Notable differences exist in the extent of decentralization, the challenges faced during implementation and inequality in access to care between the three countries. It is important for Portugal, Brazil and Pakistan to address these through reinforcing implementation strategies, tackling inequalities in access to care and enhancing monitoring and evaluation mechanism. Additionally, fostering knowledge sharing among these different countries will be instrumental in facilitating mutual learning.

## Background

Accelerating economic growth and social advancement in a country through the promotion of good governance is widely recognized as a crucial pillar of sustainable development [1]. To achieve the Sustainable Development Goals (SDGs), nations worldwide, regardless of their development status, have undertaken numerous reforms in their health systems [2]. One such reform is decentralization, which has been widely implemented across both high-income countries (HICs) and low-and-middle-income countries (LMICs) in an attempt to improve the health system performance [3]. This decentralization movement began in the latter half of the 20th century, and received strong support from the international development and donor agencies, leading to its adoption in more than 80% of countries worldwide, albeit in various degrees and forms [3, 4]. However, despite its wide adoption, empirical evidence of whether decentralization actually translates into improved health system performance remains inconclusive and controversial [5].

The term “decentralization” often means transfer of responsibilities, planning, management, decision-making authority and resources from the national to sub-national government entities (for example, regional, state and district/municipal levels) with the aim of tailoring the health system to meet local needs more effectively and responsively [6]. It is usually argued that a decentralized governance of health services bridges the gap between communities and decision-makers; improves equity, accessibility and accountability of local service providers; and fosters innovation and technical efficiency in healthcare provision [7, 8]. However, preliminary data from various countries worldwide has yielded mixed results. A recent systematic review studying the effects of decentralization in LMICs reported that decentralization of governance, financing and service delivery had positive effects on the performance of health system. Nevertheless, in the absence of adequate training and accountability mechanisms, decentralized resource management posed significant challenges and adversely affected health system performance indicators [9]. Similarly, evidence from Uganda and Indonesia suggested that, in the absence of transparent accountability mechanisms, transferring discretionary powers for resource allocation to local governments produced harmful effects on health sector [10, 11].

In contrast, some countries, including Argentina, China, India, Spain and Canada, have reported beneficial impacts of fiscal decentralization on population health, such as improved mortality rates, reduced regional disparities and higher life expectancy [12–16]. Some differences in the effects of decentralization also seem to be influenced by the income group of the country. For instance, a study examining the relationship between political decentralization and immunization coverage in 140 LMICs found that, for the low-income group, decentralized nations had higher coverage rates than centralized ones, whereas the opposite was true for the middle-income group [17]. As a result, significant disagreements and gaps persist in the assessments of decentralization conducted across different regions of the world.

It is crucial to underscore that decentralization is a complex and multidimensional phenomenon that demands thorough investigation and a deep understanding of the specific conditions within each country. These mixed findings across the literature suggest that the design and implementation of decentralization processes vary significantly and are contingent upon various contextual and socio-political factors [18]. For example, the decentralization process in Colombia unfolded gradually in phases over nearly 3 decades, whereas in other countries, such as Indonesia and Pakistan, the change happened overnight as a result of a sudden policy revolution [19–21]. Therefore, to make cross-country comparisons of decentralization’s impact on health system outcomes, it is first essential to describe and understand the process logistics, predisposing factors and implementation mechanisms in detail within the broader socio-political environment of each nation.

This research selects Brazil, Pakistan and Portugal as case studies for analysing decentralization processes within health systems. These countries have been chosen due to their varying levels of maturity and the diverse, insightful experiences each offers. Brazil’s extensive implementation of the unified health system Sistema Único de Saúde (SUS) since 1988 provides valuable insights into the long-term effects and challenges of decentralizing healthcare services, making it a pertinent case for comparative analysis. Pakistan’s inclusion is motivated by its notable decentralization efforts aimed at enhancing healthcare access, particularly in rural and underserved areas, which offers valuable lessons on decentralization dynamics in resource-constrained settings. Additionally, Portugal’s experience with decentralization reforms, including the establishment of regional health administrations, offers a contrasting perspective within the context of European healthcare systems, enabling nuanced comparisons and insights into the adaptability of decentralization strategies across diverse political and institutional frameworks. Through examining the decentralization experiences of these countries, each with distinct socio-economic backgrounds, this study aims to gain comprehensive insights into the determinants, process mechanics and challenges encountered during the planning, implementation and post-decentralization phases of this critical health sector reform. To the best of our knowledge, this would be the first narrative to compare and contrast the decentralization process and performance between LMICs and HICs. As a result, this study is expected to make a significant and valuable contribution to the expanding body of literature in this field.

## Methodology

### Research design

This study employs a literature and document review approach to comprehensively investigate the decentralization processes within the health sectors of Pakistan, Brazil and Portugal. The choice of this study design was deliberate, aiming to systematically analyse a wide range of academic/scientific papers, national and international reports, policy documents and relevant literature pertaining to decentralization within the chosen countries. By synthesizing existing knowledge and insights from various sources, this approach allows for a thorough examination of the determinants, mechanics and challenges associated with decentralization in the health sector. Through this comprehensive analysis, we aim to contribute valuable insights to the existing literature on health system governance and policy-making.

### Data collection

The primary data sources for this study included peer-reviewed academic journals, official government reports, policy documents and publications from international organizations related to health system decentralization. A comprehensive search was conducted using reputable databases such as PubMed, Google Scholar, the WHO repository and other relevant databases, covering the period up to the knowledge cutoff date in June 2023.

The initial search was carried out using relevant keywords and Medical Subject Headings (MeSH) terms in combination with Boolean operators. The MeSH terms were utilized to enhance the precision and specificity of the search.

The primary keywords and MeSH terms used for the search included “decentralization”, “health sector reform”, “health system”, “governance”, “policy implementation”, “Pakistan”, “Brazil” and “Portugal”. Synonyms and related terms were also considered to capture a wider range of relevant literature.

### Inclusion and exclusion criteria

The inclusion criteria for selecting relevant literature was based on papers and documents that specifically focused on the decentralization process in the health sector within Pakistan, Brazil and Portugal. The documents covered the planning, implementation and post-decentralization phases of the reform. Additionally, papers providing insights into the socio-political context and challenges encountered during decentralization were also considered.

In our review, a subset of strategic or explanatory documents were presented in their original language, necessitating their inclusion in the analysis. While the majority of documents were in English, a limited number were in Portuguese. To ensure comprehensive understanding, these Portuguese documents were diligently translated by native speakers proficient in both languages.

The exclusion criteria was mainly the literature not directly related to the health sector decentralization in the specified countries, and those published after the knowledge cutoff date were also excluded from the review.

### Data extraction

Information from the selected literature was systematically extracted and organized, including details on the decentralization timeline, implementation strategies, mechanism for resource allocation, accountability mechanisms, challenges and any reported health system performance indicators. Key findings and insights related to the decentralization experience of each country were documented.

### Data analysis

A thematic analysis approach was employed to categorize and synthesize the extracted data. Key themes and patterns related to the determinants, mechanics and challenges of decentralization were identified. Additionally, the performance outcomes reported in the literature were compared and contrasted between the developed and developing countries to draw relevant conclusions (Table 1).

**Table 1:** Comparison of decentralization processes and health system reforms across Pakistan, Portugal and Brazil

Theme	Pakistan	Portugal	Brazil
Country profile	Population: 232 million GDP: 376.53 billion US$ Health expenditure: 1.2% of GDP	Population: 10 million GDP: 249.89 billion US$ Health expenditure: 10.6% of GDP	Population: 203 million GDP: 1.92 trillion US$ Health expenditure: 3.8% of GDP
Pre-decentralization	Health functions primarily centralized; limited provincial involvement in health functions	Establishment of regional health administrations; municipalities empowered in primary care	Health services mix, including public and private; INAMPS centralized public health assistance
Decentralization timeline	2009: change in resource allocation 2010–2011: unanimous passing of 18th constitutional amendment	Since 1976: ongoing decentralization process integrated with constitution	Early 1990s: establishment of SUS
Implementation strategies	Devolution: transfer of functional, jurisdictional and fiscal responsibilities to provinces (2010–2011)	Administrative regionalization: creation of regional health administrations (1993) Municipalities assigned responsibilities for managing, constructing and maintaining primary healthcare centres (1999); implementation of PPP contracts for hospital management	Devolution: shift of responsibilities to municipalities and states Concept of decentralization introduced through Federal Constitution of 1988, further formalized by Law no. 8080/1990, establishing SUS; transition to decentralized management model guided by regulatory norms such as NOBs and NOAS (1990s and early 2000s)
Mechanism for resource allocation	Equity-based distribution of resources; larger share (56–58%) to provinces	Central government holds responsibility for funding and regulation	Constitutional amendment mandating revenue allocation for healthcare
Accountability mechanisms	Provinces gain autonomy in planning and decision-making; establishment of Ministry of National Health Services Regulation and Coordination	RHAs responsible for regional health planning and coordination	Tripartite and bipartite commissions facilitating shared decision-making Establishment of health councils as platforms for social participation; introduction of health conferences and councils for ongoing system refinement
Challenges	Abrupt and inadequately planned devolution process; weak institutional capacities	Slow and constrained decentralization process; limited impact on higher levels of healthcare services	Regionalization challenges; heterogeneous results across regions
Post-decentralization	Increased provincial autonomy; each province formulated its own context-specific health policy; opened doors for direct engagement with international donor agencies; implemented essential service delivery reforms at the district level Limited effectiveness; limited evaluation of decentralization impact; challenges in institutional capacities and budget	Slow decentralization, focused on primary care; heterogeneous results in regionalization; funding stability secured through constitutional amendment; municipalities granted competencies for health management	Transition to decentralized management model during 1990s and early 2000s; significant shift towards local autonomy in healthcare management guided by regulatory norms; constitutional amendment for stable funding base Implementation of regionalization as complementary approach to decentralization, regulated through NOAS and reinforced by Pact for Health
Improvements in the health system	Improved health indicators: decrease in infant and under-5-year-old mortality rates	Notable improvements in access and utilization of healthcare services	Positive health outcomes: decrease in infant and under-5-year-old mortality rates

*INAMPS* National Social Security Healthcare Institute; *NOAS* Operational Norms of Health Assistance; *NOB* Norms of Basic Operationalization; *RHA* regional health administration; *SUS* Sistema Único de Saúde

### Pakistan – country profile

Pakistan, situated in the South Asian region and bordered by India, China, Afghanistan, Iran and the Arabian Sea, stands as the fifth most populous country globally, with a current population of 232 million, projected to increase to 281 million by 2030 [22]. Encompassing an area of 881 913 km^2^, Pakistan ranks as the 33rd-largest country and maintains a population density of 302.08 people/km^2^. The country is divided into four provinces: Punjab, Sindh, Khyber Pakhtunkhwa (KPK) and Baluchistan. In addition, there are three federating units: Azad Jammu and Kashmir (AJK), Gilgit Baltistan (GB) and Islamabad Capital Territory (ICT) [23]. Currently, Pakistan has a gross domestic product (GDP) of 376.53 billion US$, gross national income (GNI) per capita of 1580 US$, and a GDP growth rate (annual %) of 6.2. As per the World Bank’s country classification, Pakistan is classified as a lower-middle-income economy [24]. The government current expenditure on health accounts for 1.2% of the total GDP.

### Pre-decentralization scenario

Pakistan’s health system is characterized by a mixed structure, comprising various vertical and horizontal institutions, including the public sector, private sector, parastatal organizations, philanthropists and donor agencies. Health services are organized into a three-tiered delivery system, encompassing primary, secondary and tertiary levels of care. Prior to the decentralization, the constitution contained two legislative lists: the federal legislative list (FLL) and the concurrent legislative list (CLL), which determined the division of legislative authorities between the federal and provincial governments. Under this setup, the central government had exclusive power to formulate laws with respect to any matter listed in the FLL while both the central and provincial governments had the authority to make laws related to matters in the CLL. Matters not specified in either list fell under the jurisdiction of the provincial assemblies. Consequently, a majority of health functions fell within the jurisdiction of the federal ministry of health (MoH). The MoH assumed several stewardship responsibilities such as planning and policy formulation, service delivery programming, health financing, coordination with international agencies, human resource management and drug regulation and implementation of the 11 vertical health programs, as well as monitoring and evaluation of health services. Although the provincial governments played a co-financing role in most of these important health functions, their involvement was primarily administrative and passive in nature [25].

### The decentralization reform

Decentralization is often classified into three forms:

1. Deconcentration (equivalent to opening a branch office in another region);
2. Delegation (transferring certain responsibilities to a sub-national government);
3. Devolution (a complete take-over of functions and authority from the central government by a lower tier) [26].

In Pakistan, the decentralization reform was preceded by a change in resource allocation formula for the federal and provincial authorities in 2009. This led to the adaptation of an equity-based distribution of resources, with a larger share (56–58%) going to the provinces based on their developmental requirements and local challenges [27, 28]. Subsequently, the unanimous passing of the 18th constitutional amendment by all political parties in 2010–2011 marked a radical, politically driven decentralization process, implemented in its strongest form – devolution [29, 30]. As a result, 17 federal ministries, including health to the provinces, were dissolved, transferring functional, jurisdictional and fiscal responsibilities to the four sub-national governments [31]. The amendment also led to the abolition of the CLL, granting provinces greater autonomy in exercising full administrative and financial control over their health systems [30, 32].

However, the devolution process was abrupt and inadequately planned. Provinces lacked prior experience in independently assuming stewardship functions (such as budgeting and policy formulation), leading to a chaotic situation [21]. Moreover, with the abolition of the CLL, many of the functions previously being managed by the MoH were dispersed across various federal institutions, including the Economic Affairs Division, Ministry of Inter Provincial Coordination, Federal Bureau of Statistics and Capital Administration and Development [21]. Due to the demolition of a central governing body, several difficulties were raised in managing national and international coordination for global health commitments, licensing of medical and paramedical practitioners and health regulation, particularly drug regulation and licensing. As a result, a recentralization process was initiated within 2 years to consolidate the dispersed central functions under a single federal entity. In 2013, the new federal ministry named Ministry of National Health Services Regulation and Coordination (MoNHSR&C) was quickly reconstituted the basis of recommendations of a multi-donor WHO mission [30]. The federal government assumed responsibilities for managing international trade and agreements, drug pricing and licensing, human resource regulation, surveillance of ports and borders and co-financing important vertical health programs [33].

### Post-decentralization – progress and challenges

After the devolution, the provinces in Pakistan took several planning and governance initiatives to fulfil their new stewardship role in the health sector. For the first time, each province formulated its own context-specific health policy and action plan, outlining a strategic direction for the sector for the next 10 years. This also opened doors for the international donor agencies to engage directly with the provinces in alignment with their provincial priorities. Moreover, two provinces, Sindh and KPK, undertook district-level planning to implement essential service delivery reforms [21].

However, despite these efforts, the implementation of the newly formed health sector strategies remained limited and ineffective due to weak institutional capacities and insufficient budgets [21]. A comprehensive review by Zaidi et al. in 2018 revealed a significant increase in healthcare budgetary spending across all provinces, with a greater portion of the increased health spending being allocated to salaries and administrative costs [21]. Delayed release of budgetary transfers from the federal government also hindered the effective functioning of vertical health programs at the provincial level [21, 34]. Nonetheless, provinces managed these challenges by generating their own resources or seeking additional support from external donor agencies.

Provinces also made substantial progress in improving the governance and delivery of the primary healthcare through multiple reforms and innovations. Initiatives included implementing public–private partnership (PPP) models at the district level, contracting out of primary healthcare services to private providers and integrating overlapping vertical health programs [21, 35, 36]. Despite these positive steps, the influence of local bureaucracies and the political influence of provincial elites posed some serious challenges in maintaining merit and transparency across all levels of provincial health management.

Despite the shortcomings in planning and execution, the devolution in Pakistan did result in increased ownership of healthcare by provincial stakeholders and bureaucrats. Moreover, the devolution fostered a sense of healthy competition among all four provincial governments, driving them to deliver better healthcare and to gain increased political support from their communities. Hence, this transition to a decentralized health system, though abrupt, laid the groundwork for several legislative and innovative reforms at the provincial health level.

While no formal evaluation to measure the impact of the decentralization reform in Pakistan has been conducted thus far, the country’s health indicators have shown considerable improvement over time, potentially attributable to the health sectors reforms and initiatives undertaken by local governments following devolution. For instance, the infant mortality rate in Pakistan decreased from 74 per 1000 live births in 2012 to 62 per 1000 live births in 2018. Likewise, the under-5-year-old mortality rate also exhibited a reduction from 89 per 1000 live births in 2012 to 74 per 1000 live births in 2018 [37]. However, it is strongly recommended that a systematic evaluation of the health system indicators be conducted in future to ascertain the actual impact of decentralization on the health sector performance and efficiency in Pakistan. Such an evaluation would provide valuable insights into the successes and challenges of the decentralization process and help inform further improvements in the country’s healthcare system.

### Portugal – country profile

Portugal is a coastal country located in the Southern European region. The mainland, located in the southwest of the Iberian Peninsula, shares its borders with Spain to the north and east, while being bordered by the Atlantic Ocean to the west and south. In addition to the mainland, Portugal includes two autonomous regions located in the Atlantic Ocean: the archipelagos of Azores and Madeira. In terms of territory, Portugal covers a total area of 92 212 km^2^ and has a population of approximately 10 million habitants resulting in a population density of 112.6 habitants/km^2^. The country is administratively divided into seven regions: North, Centre, Lisbon Metropolitan Area, Alentejo, Algarve, Azores and Madeira [38, 39].

In 2021, Portugal’s GDP stood at 249.89 billion US$, with an annual growth rate of 4.9%. The gross national income (GNI) per capita based on purchasing power parity was of 35 470$, experiencing an annual growth rate of 4.5% [40]. Regarding healthcare, the government’s current expenditure on health in 2022 amounted to 10.6% of the total GDP [41].

### The decentralization reform

Since 1976, Portugal has been undergoing an ongoing decentralization process as an integral part of the constitution of the Portuguese Republic. The Portuguese health system was established in 1979, evolving from the integration and complementarity of various response levels, including primary, hospital, continuing and palliative care. This health system is characterized by the coexistence of three distinct systems: the national health system (NHS), public and private insurance schemes for certain professions (health subsystems) and private voluntary health insurance. The healthcare delivery system in Portugal comprises a network of public, social and private healthcare providers. The Ministry of Health holds the responsibility for developing health policies, with its principal function centred on the planning, regulation and management of the NHS [42, 43]. Through this decentralized approach, Portugal has established a healthcare system that integrates multiple sectors and strives to provide comprehensive care to its population.

The principles for the organization and functioning of the health system in Portugal were introduced by the 1990 Basic Law on Health. According to this law, the NHS would be supervised by the Minister of Health and administered at the region health level by the respective regional health administrations (RHAs). A significant milestone in the Portuguese health system occurred in 1993 when administrative regionalization was established, leading to the creation of five RHAs: North, Centre, Lisbon and Tagus Valley, Alentejo and Algarve [44].

The RHAs played crucial roles in the healthcare system, including:

1. Developing activity and budget plans for the NHS in their respective regions;
2. Representing the NHS at the regional level;
3. Guiding, coordinating and monitoring the regional areas of the NHS;
4. Regulating demand among healthcare establishments and services in the region and overseeing their operations;
5. Contracting with private healthcare providers to offer healthcare services to NHS beneficiaries;
6. Coordinating patient/beneficiaries’ transport to ensure accessibility to healthcare services.

Through the establishment of RHAs, Portugal aimed to enhance regional coordination and management of healthcare services, bringing healthcare decision-making closer to the local level and promoting more efficient and effective healthcare delivery across different regions.

In 1999, a new law was introduced in Portugal that assigned new health functions to municipalities. These functions included the management, construction and maintenance of primary healthcare centres. The aim was to decentralize certain responsibilities to the local level and empower municipalities to play a more active role in the provision of primary healthcare services. However, despite the enactment of this legislation, it was not fully implemented in the subsequent years. As a result, the intended decentralization and involvement of municipalities in managing primary healthcare centres did not materialize to its full extent, and the status quo in health service management may have remained largely unchanged at the time.

The implementation of various laws and reforms in Portugal led to significant changes in the decentralization process of the health system. As a result, each regional health administration (RHA) gained local influence and assumed a central role at the regional level. The management of hospitals, primary healthcare centres and other health institutions came under the authority of the RHAs, enabling them to make agreements with the private sector and develop health plans on the basis of the specific needs of the population in their regions [43].

While hospitals remained public property, a different management model was introduced through PPP contracts. These contracts ensured the construction and maintenance of hospital infrastructures, as well as the provision of healthcare services. This approach aimed to improve the efficiency and quality of healthcare delivery by involving private sector expertise while still upholding the public ownership of healthcare facilities [42]. Furthermore, with the decentralization process, managers of health institutions were locally recruited, giving considerable power and autonomy to the new regional boards. This local management approach allowed for a more tailored and responsive decision-making process, as managers were well-acquainted with the specific health needs and challenges of their respective regions [43].

In 2019, a significant development occurred in Portugal’s health system with the approval of a new Basic Law on Health (Lei no. 95/2019 de 4 de setembro). This law marked a shift in the organization of the NHS towards regionalization and decentralized, participative management [45]. Furthermore, in 2019, the framework for the transfer of competencies to municipal bodies and intermunicipal organizations in the health domain was completed. This transfer of competencies aimed to further strengthen the role of local municipalities in the provision and management of healthcare services. By empowering municipal bodies and intermunicipal organizations, the health system aimed to foster more responsive and locally tailored healthcare solutions. Under this framework, municipal authorities in Portugal were granted several competencies that further reinforced their role in the health domain. Municipalities were empowered to take proactive measures to promote health and well-being within their communities, implementing programs and campaigns to raise awareness about healthy lifestyles and disease prevention. Additionally, they were tasked with fostering initiatives to support active ageing and improve the quality of life for elderly citizens. Another crucial aspect of the competencies granted to municipalities was the responsibility for maintaining healthcare buildings and facilities, ensuring that the infrastructure remained in optimal conditions to deliver quality healthcare services to the public. Furthermore, the law emphasized the importance of involving municipalities in shaping local health policies, allowing them to contribute valuable insights based on their unique understanding of the health needs and priorities of their respective populations. By entrusting municipalities with these competencies, the health system sought to foster a more community-centred approach to healthcare, promoting inclusivity and empowering local authorities to play an active role in the well-being of their citizens.

In addition to the Basic Law on Health, other legislative resolutions have been implemented in Portugal to further advance the health decentralization process. The 21st Government Program played a significant role in this regard by emphasizing the importance of democratic decentralization of public administration as an essential state reform, aligning with the principles enshrined in the Portuguese constitution (no. 1 of the 6th article).

In theory, decentralization has been incorporated into the constitutional and legal frameworks of the NHS in Portugal over the past decades. Proposals for devolution and delegation of authority from the central government to regional units have been put forward. However, in practice, the responsibility for planning and resource allocation in the Portuguese health system has remained highly centralized. The administrative devolution has not fully materialized, leading to a gradual decentralization process that primarily focuses on bringing healthcare services closer to citizens and addressing their specific needs. This process has been predominantly centred around primary healthcare.

While RHAs have been granted some autonomy, particularly in budget setting and spending, their jurisdiction has largely been limited to primary care. Most public hospitals still fall under the central administration, and their financial resources primarily come from the state budget. Decisions regarding staff allocation, technical guidance and organizational rules for these hospitals are also predominantly made at the central level [46].

As a result, the decentralization process in the Portuguese health system has been slow and constrained, with limited impact on the management and decision-making processes at higher levels of healthcare services. The focus has primarily been on strengthening primary healthcare services and improving accessibility and quality of care at the local level. However, achieving a more comprehensive and fully decentralized health system remains a challenge due to the prevailing centralization of resources and decision-making authority in the hospital sector.

It is anticipated that, by the end of 2023, the RHAs will be eliminated, marking a significant step towards complete devolution of authority from the central government to local health units (LHUs). These LHUs are envisioned to encompass a comprehensive range of health services, including primary care, hospital care, palliative and continuing care and public health initiatives. With the elimination of RHAs, LHUs are expected to assume greater autonomy in managing the majority of health competences. This shift will foster a close and collaborative partnership between LHUs and municipal bodies, allowing for more localized decision-making and tailored health policies that reflect the specific needs and priorities of different communities. This complete devolution of authority to LHUs is anticipated to lead to a more decentralized and citizen-centred health system in Portugal, with a focus on delivering responsive, efficient and equitable healthcare services across the nation.

The process of decentralization of health in Portugal is a dynamic and ongoing endeavour. As the country continues to implement changes in its health system organization and management, there is a pressing need for further research and evaluation. To assess various dimensions such as effectiveness, efficiency, access, patient-centredness safety and equity, this is crucial to understanding its impact on the health system’s overall performance.

### Brazil – country profile

Brazil is a vast federative republic situated in South America, covering an area of 8.5 million km^2^, making it the fifth-largest country globally. With a population estimated at 203 million in 2022, and an annual growth rate of 0.52%, Brazil ranks as the seventh most populous nation in the world, with population density of 23.86 people/km^2^. The political structure of Brazil is organized into three levels of government: the federal government, 26 states/provinces and one federal district, and 5563 municipalities. Currently, Brazil’s GDP is about 1.92 trillion US$, GNI per capita is 8917.7 US$, and GDP growth rate is +2.9% annually [according to the Brazilian Institute of Geography and Statistics (IBGE)]. The country’s government consumption expenditure on health stands at around 3.8% of the GDP, while the total consumption expenditure on health amounts to 9.6% of the GDP, with a significant portion spent by households and non-profit institutions serving households. With respect to its economic status, Brazil is classified as an upper-middle-income economy by the World Bank.

### Pre-decentralization scenario

The decentralization process of the health system in Brazil occurred during the establishment of the national public health system, called the Sistema Único de Saúde (SUS), in the early 1990s. Prior to the creation of SUS, the Brazilian health system comprised a mix of health services established at different periods, including public and private services, and philanthropic institutions. At that time, public health assistance in Brazil was the responsibility of the Social Security and Assistance Ministry (MPAS), primarily provided through the National Social Security Healthcare Institute (INAMPS), which was created in 1977 to centralize various public health services [47].

The Ministry of Health, in contrast, was primarily focused on epidemiological vigilance, sanitary control and collective health actions, and was relatively underfunded compared with the INAMPS. The assistance services provided by the INAMPS were accessible only to workers with formal employment and their dependents, who received care through public hospitals and ambulatory centres, as well as private services contracted by the state. Meanwhile rural workers, informal labourers and unemployed citizens relied on philanthropic institutions for their healthcare needs.

During the 1970s and the 1980s, Brazil witnessed significant progress in health coverage supported by funding from the federal government [48]. This funding was directed not only towards the private sector through contracting private services but also towards expanding public health initiatives and actions. Initiatives such as the Integrated Health Actions (AIS) and the Program of Interiorization of Health and Sanitation Actions (PIASS) were introduced, promoting regionalization and establishing a hierarchy between municipalities, states and the federal government [47]. The AIS and PIASS were integral to the maturation of the public health debate in Brazil, paving the way to the Sanitary Reform Movement. This movement played a crucial role in formulating both the theoretical and political basis for the establishment of the SUS in 1990. The creation of the SUS and the decentralization process in Brazil aimed to address the issue of centralized resources and power within the federal government. Furthermore, the new health system sought to transform the existing health model, which heavily emphasized hospitalization and disease treatment, into one that prioritized prevention and health promotion actions. Recognizing the importance of preventive measures in improving overall population health, the SUS aimed to shift the focus from solely providing curative care to actively promoting wellness and disease prevention. This change in approach was deemed essential for achieving better health outcomes and ensuring a more sustainable and efficient health system for the Brazilian population [49].

### Decentralization

The concept of decentralization, involving the sharing of management and decision-making power with the municipalities and states, was fundamental guiding principle stated inside the Health section of the Federal Constitution of 1988. This principle was further formalized and solidified with the approval of Law no. 8080/1990, which officially established the SUS in 1990. The process of decentralization and the establishment of the SUS occurred gradually during the early years of the 1990s, in line with the prevailing political and governmental priorities.

Indeed, Brazil’s decentralization process within the health sector can be considered a devolution process. In this context, subnational governments, including municipalities and states, not only assume responsibility for providing healthcare services but also gain control over the planning and decision-making aspects of health policies [50].

The SUS Organic Law (Law no. 8080) outlines a clear division of competencies among different levels of government in Brazil’s health system. According to this law, municipalities have the responsibility to manage and provide public health services to their local populations. In contrast, states and the federal government play a role in coordinating, evaluating and participating in the formulation of health policies. Their specific tasks include areas such as health vigilance, epidemiology, sanitation, health norms and financial cooperation [51].

An important feature of the decentralization process in Brazil’s health system was the establishment of tripartite and bipartite commissions. These commissions include representatives from the federal, state and municipal governments, and they serve as platforms for shared decision-making on health policies. This collaborative approach ensures that decisions are made collectively, taking into account the perspectives and needs of all levels of government. Additionally, health conferences and councils were introduced as mechanisms for social participation. These platforms allow citizens and civil society organizations to actively engage in the policy-making process, providing valuable input and feedback on health issues [52].

The process of decentralization in Brazil’s health system during the 1990s was characterized by different phases, influenced by changes in the norms that governed the transition to a decentralized management model. These norms, known as the Norms of Basic Operationalization (NOB), were introduced in 1991, 1993 and 1996, respectively. The NOBs established terms and guidelines for local governments to adhere to the management transition plan in various stages, based on their capacity and willingness to take on responsibilities, while adhering to the structural and organizational conditions specified by these norms [49, 51].

A significant aspect of the NOBs was the requirement for every level of government to establish health councils, with representatives of users occupying at least half of the seats. This emphasis on social participation through health councils underscores the priority given to involving citizens and civil society in the formulation of health policies, as mandated by the regulatory laws of the SUS. By ensuring that health councils have a significant user representation, the goal was to foster greater transparency, inclusivity and responsiveness in decision-making processes related to health services. The effects of decentralization were felt as an important increase in the participation of municipalities during the 1980s and the 1990s in health actions. For example, the percentage of health facilities managed by the municipalities rose from 22% of the facilities in 1981 to 69% in 1992. In terms of employment, the municipalities, which were responsible for employing 16% of the public health workers in 1981, rose to 44% of the total amount of workers inside the public health system in 1992 [51].

### Post-decentralization

Over its more than 30-year trajectory, the Brazilian SUS has undergone significant changes and adaptations, particularly in terms of funding and organization. The decentralization guidelines, established by regulatory norms such as the NOB and the Operational Norms of Health Assistance (NOAS) played a crucial role in inducing the transition to a decentralized model during the 1990s and early 2000s,

The NOBs and NOAS provided guidelines and incentives for municipalities to take on greater responsibilities in managing their health systems. As a result of these efforts, by the year 2000, an impressive 99% of Brazilian municipalities were fully (9.4%) or partially in charge of the management of their health systems. This marked a significant shift towards greater local autonomy and ownership in the delivery of healthcare services. The funding system of SUS has seen continuous improvement through the same norms, incorporating new sets of rules to define financing to municipalities and states [53].

In the 2000s, a significant step was taken to ensure adequate financing for the health system. A constitutional amendment was approved, mandating that states and municipalities allocate a minimum of 12% and 15% of their yearly revenue, respectively, to the health system. Meanwhile, the federal government’s minimum allocation was defined on the basis of the previous year’s health expenditure, adjusted for inflation and GDP growth. This amendment aimed to secure a stable and sufficient funding base for the SUS, promoting financial sustainability and stability.

The impact of these funding measures has been substantial. Between 1989 and 2014, real health expenditure per capita in Brazil increased by an impressive 149%. This growth was divided between the public sector, which contributed 46% of the increase, and the private sector, which contributed the remaining 54%. The increased investment in the health system has led to significant improvements in healthcare access, infrastructure and services, benefiting both the public and private sectors [52].

Regionalization emerged as a crucial aspect of the public health agenda in Brazil during the late 1990s and early 2000s. It was seen as a complementary approach to decentralization, aiming to address fragmentation and improve the delivery of healthcare services by optimizing available resources through regional networks with shared planning and programming. Official regulation of regionalization was introduced through NOAS in 2001 and was further reinforced by the Pact for Health in 2006.

The implementation of regionalization, however, has yielded heterogeneous results across different regions of the country. Some regions have made significant advances in successfully executing regionalization, leading to improved service integration, resource utilization and healthcare outcomes. However, certain regions have faced challenges in fully executing regionalization [54].

Overall, since the implementation of the decentralized and primary-care-based SUS, there have been notable improvements in the delivery of healthcare services, particularly through the Family Health Strategy (PSF) [55]. The SUS has contributed to better health outcomes for the population. In terms of access and utilization of health services, significant progress has been observed. From 1998 to 2013, the percentage of the population that had visited a doctor within the last 12 months increased from 54.7% to 74.2%. These figures demonstrate improved access to healthcare services and a greater utilization of medical care among the Brazilian population.

Moreover, health outcomes have seen remarkable advancements. The infant mortality rate has significantly decreased from 53.4 per 1000 live births in 1990 to 14 per 1000 live births in 2015. Similarly, the under-5-year-old mortality rate has also declined from 64.2 per 1000 live births in 2012 to 15.7 per 1000 live births in 2015 [52].

Although there is evidence that the decentralized SUS has contributed to these positive health outcomes, it is important to acknowledge that other factors have also played a role in the improvements. These factors include broader societal advancements and public health initiatives, such as improved access to water and sanitation, enhanced immunization coverage and other public health initiatives, making it challenging to isolate the specific effects of the decentralization process alone.

As such, the specific impact of decentralization on the health system in Brazil remains a subject of debate and requires further investigation. Future research should aim to disentangle the various factors that have contributed to the observed improvements in health outcomes, providing a more nuanced understanding of the role of decentralization in shaping Brazil’s healthcare landscape [56].

## Discussion

Decentralization involves transferring technical, managerial and financial responsibilities from the centre to the periphery, such as provinces or districts [57]. It is advocated at the top level as an essential element for achieving universal health coverage [58]. In theory, decentralization offers several benefits, including faster decision-making at the local level, consideration of local customs and perspectives and a more responsive health system. However, successful implementation relies on strong democratic values, accountability systems and professional standards within the local governments.

In this study, we examined the experiences of three countries from different continents with distinct socioeconomic profiles, each having unique experiences in implementing and advancing decentralization. Our aim was to assess the impact of this bold political move on the health of the population in these countries, evaluating both the positive and negative aspects. In Pakistan, the implementation of decentralization faced challenges common to other LMICs. Issues at the local level included inadequate technical skills, ineffective delegation of decision-making power and a lack of local interest. A rapid implementation led to coordination problems between central and local authorities, particularly during political conflicts. Nevertheless, positive effects on the health system were observed, such as improvements in financing, governance and service delivery. Studies from Pakistan have also reported an increase in community participation after decentralization, as it required more engagement from the community in healthcare planning [59]. Decentralization reforms in Pakistan prioritized health, leading to increased investment in the sector and granting local decision-making power, resulting in more responsive and effective healthcare services. Local managers are better adapted to local needs and demands, leading to better-quality care.

When comparing Portugal to other European countries it becomes evident that decentralization efforts have been undertaken in several countries across the continent. Countries such as Sweden, Norway, Finland and Denmark have embraced decentralization as a general principle in their health systems to achieve efficiency and improved healthcare outcomes [60]. Similarly, the United Kingdom has decentralized its health system, granting devolved powers to regions such as Wales, Northern Ireland and Scotland, enabling them to manage and deliver healthcare services according to their specific needs [61].

In Southern European countries such as Italy, Portugal and France, decentralization has also been implemented to a lesser extent, and the impact varies. It is essential to acknowledge that each country has its own unique context, political structure and healthcare system, influencing the success and outcomes of their decentralization efforts. Portugal’s decentralization process started a decade later than other European countries and has been relatively slow [46]. The intention was to enhance access to healthcare services, improve quality of care and optimize resource utilization [43]. However, potential drawbacks, including service fragmentation, unequal access to care, lack of standardization and increased bureaucracy, need to be addressed to ensure high-quality and accessible healthcare services [62]. As of now, the decentralization in Portugal does not cover all domains, with most public hospitals still belonging to the central administration [63].

Brazil, in contrast, has implemented successful decentralization reforms in its health system since the establishment of SUS in the 1990s [64]. These reforms aimed to transfer decision-making authority to sub-national levels, focusing on financing, management and inter-managerial agreements. This decentralization has facilitated the provision, financing, management and regulation of a wide range of health services, with substantial collaboration between all three levels of government. However, challenges remain, including disparities in services and quality across the country, unstable funding streams and expenditure allocation not fully based on the level of need [64]. Other South American countries have also adopted decentralization reforms to improve health outcomes and increase local decision-making power, albeit with variations in approaches depending on their unique contexts and healthcare systems [65].

Although decentralization reforms have achieved some success, challenges persist in their implementation. These challenges include capacity-building, resource limitations and resistance to change. Decentralization can also lead to inequality in access to care, with some regions having better access to healthcare services than others. This can lead to disparities in health outcomes, fragmented services and inefficiencies in care coordination across different levels of care [66].

Comparing all three countries, it is evident that all three have prioritized health in their decentralization reforms and aimed to enhance local decision-making power. Brazil has made significant progress in implementing decentralization reforms, while Portugal and Pakistan are still in the process of doing so. The decentralization context of these three countries holds significant relevance beyond their respective borders, offering valuable insights and lessons that can inform decentralization efforts elsewhere. Despite their diverse socioeconomic backgrounds and healthcare systems, these countries share common challenges and successes in their decentralization journeys. The experiences of Pakistan highlight the complexities and implementation challenges faced by many LMICs striving to devolve power and resources to local levels. Portugal’s slow but deliberate approach underscores the importance of addressing potential drawbacks, such as service fragmentation and unequal access, to ensure the success of decentralization initiatives. Brazil’s remarkable progress in decentralization, particularly in the health sector, serves as a beacon of success for countries seeking to improve service delivery and governance through greater local autonomy. The extent, progress and challenges in the decentralization processes vary among the three countries, each requiring ongoing evaluation and improvement to achieve the desired outcomes. By analysing and synthesizing the decentralization experiences of these countries, policy-makers and stakeholders worldwide can gain valuable insights into the contextual factors, implementation strategies and accountability mechanisms that contribute to successful decentralization reforms.

The study may be limited by the availability and scope of the literature on health sector decentralization in the selected countries. Additionally, the data used in this review will be up to the knowledge cutoff date, and any subsequent developments or changes in the decentralization strategies may not be captured.

## Conclusion

In conclusion, all three countries have undertaken decentralization reforms in their health systems with the shared goal of improving health outcomes and empowering local decision-making. However, notable differences exist in the extent of decentralization, the challenges faced during implementation and inequality in access to care between the three countries. Thus, additional research is needed to obtain more rigorous evidence that can allow to learn from different experiences in LMIC and HICs countries.

## Way forward

Moving forward, it is important for Portugal, Brazil and Pakistan to address the challenges encountered during their decentralization processes. This can be achieved through reinforcing implementation strategies, tackling inequalities in access to care, and enhancing monitoring and evaluation mechanism. Additionally, fostering knowledge sharing among these different countries will be instrumental in facilitating mutual learning.

By proactively addressing these aspects, Portugal, Brazil and Pakistan can continue refining and strengthening their decentralization efforts in the health sector. This, in turn, will help reduce disparities in healthcare, improve accessibility to services and ultimately lead to better health outcomes for their respective populations. Collaborative efforts and continuous improvement will be essential in achieving the overarching goal of equitable and high-quality healthcare for all.

## Data Availability

Not applicable.

## Abbreviations

AIS: Integrated Health Actions
AJK: Azad Jammu and Kashmir
CLL: Concurrent legislative list
FLL: Federal legislative list
GB: Gilgit Baltistan
GDP: Gross domestic product
GNI: Gross national income
IBGE: The Brazilian Institute of Geography and Statistics
ICT: Islamabad Capital Territory
INAMPS: National Social Security Healthcare Institute
KPK: Khyber Pakhtunkhwa
MPAS: Social Security and Assistance Ministry
NHS: National health system
NOAS: Operational Norms of Health Assistance
NOB: Norms of Basic Operationalization
PIASS: Program of Interiorization of Health and Sanitation Actions
PPP: Public–private partnerships
PSF: Family health strategy
RHA: Regional health administration
SUS: Sistema Único de Saúde
